# Comparative Transcriptome Analysis Reveals Significant Differences in MicroRNA Expression and Their Target Genes between Adipose and Muscular Tissues in Cattle

**DOI:** 10.1371/journal.pone.0102142

**Published:** 2014-07-09

**Authors:** Jiajie Sun, Bowen Zhang, Xianyong Lan, Chunlei Zhang, Chuzhao Lei, Hong Chen

**Affiliations:** 1 College of Animal Science and Technology, Northwest A&F University, Shaanxi Key Laboratory of Molecular Biology for Agriculture, Yangling, Shaanxi, China; 2 Institute of Cellular and Molecular Biology, Jiangsu Normal University, Xuzhou, Jiangsu, China; George Washington University School of Medicine and Health Sciences, United States of America

## Abstract

The posttranscriptional gene regulation mediated by microRNAs (miRNAs) plays an important role in various species. However, to date limited miRNAs have been reported between fat and muscle tissues in beef cattle. In this paper, 412 known and 22 novel miRNAs in backfat as well as 334 known and 10 novel miRNAs in longissimus thoracis were identified in the Chinese Qinchuan beef cattle. Bta-miR-199a-3p, -154c, -320a and -432 were expressed at higher levels in backfat tissue, while bta-miR-1, -133a, -206, and -378 were also significantly enriched in muscle tissue. Functional analysis revealed that fat-enriched miRNAs targeted *PRKAA1/2*, *PPARA* and *PPARG* genes to modulate lipid and fatty acid metabolism, and muscle-enriched miRNAs targeted *CSRP3* gene to present function involved in skeletal and muscular system development. The results obtained may help in the design of new selection strategies to improve beef quality.

## Introduction

miRNAs are single-strand RNA molecules of ∼22 nucleotides in length [1], which play a crucial role in developmental processes by regulating the expression of target transcripts [2]. To date, thousands of miRNAs have been discovered, and they have become one of the most abundant categories of gene regulatory molecules in mammalian species [3]. It is estimated that about 30% of all protein-coding genes are regulated by miRNAs [4].

The majorities of miRNAs are conserved across species and play essential roles in regulating many distinct processes. In myocyte proliferation and differentiation, miR-206 was the first miRNA showed to play an important role in skeletal muscle development by regulating the expression of connexin43 in C2C12 cells, a gap junction protein required for skeletal myoblast fusion [5]. In addition, the miR-206 gene is induced by MyoD, which promotes myogenic differentiation [6]. Bone Morphogenetic Protein-2 (BMP-2), which is known to inhibit myogenesis, represses the expression of miR-206 by inhibiting its maturation process [7]. MiR-1 and miR-133, as well as miR-206, are all muscle specific miRNAs [8]. MiR-1 and miR-133 have distinct roles in modulating skeletal muscle proliferation and differentiation in cultured myoblasts *in vitro* and in *Xenopus laevis* embryos *in vivo* [9]. Similarly, overexpression of miR-181 during muscle differentiation is important to promote myogenesis by down-regulating the homeobox protein Hox-A11, an inhibitor of muscle differentiation [10]. MiR-486 has also been shown to induce myoblast differentiation by down-regulating Paired-box-containing 7 (Pax7) [11], while MiR-27b regulates Paired-box-containing 3 (Pax3) protein levels and ensures myogenic differentiation [12]. In lipid and fatty acid metabolism, the first report suggested that miR-14 were involved in fat metabolism [13]. Deletion of mir-14 resulted in animals with increased levels of triacylglycerol and diacylglycerol, whereas increases in mir-14 copy number had the converse effect. Another earlier study demonstrated that miR-143 is involved in adipocyte differentiation and may act through target gene extracellular-regulated protein kinase 5 (*ERK5*) [14]. Furthermore, miR-8/miR-200 family promotes adipogenesis by inhibiting Wnt signaling, which is a negative regulator of adipogenesis [15]. So far, the miRNAs have been indentified to play crucial roles in muscle and adipose development, but the underlying mechanisms for the changes observed in these tissues remain largely unknown, which warrants further studies.

Despite the recognized importance of miRNAs in regulating gene expression, there has been little information about miRNAs expression in cattle. Recently, related studies presented in bovine species have been conducted to provide insight into the miRNAs population by investigating the characteristics, expression pattern and features of their target genes. For instance, 59 distinct miRNAs were identified from bovine adipose tissue and mammary gland [16]. Bta-mir424 and bta-mir-10b are highly abundant in germinal vesicle oocytes, as well as in early stage embryos (until 16-cell stage) [17]. MiR-196a is a bona fide negative regulator of the newborn ovary homeobox gene (*NOBOX*) during bovine early embryogenesis [18]. Expression of bovine nucleoplasmin 2 (*NPM2*) is temporally regulated during early embryogenesis by miR-181a [19]. Approximately 20% of the miRNAs involved in adipogenesis and lipid deposition were identified as being correlated with backfat thickness [1].

Given the emerging roles of miRNAs in fat and muscle development, identifying the expression pattern of miRNAs is an important step to investigating the function of miRNAs in the course of bovine muscle development and lipid metabolism and adipogenesis. In the present study, we therefore used Illumina sequencing technology to characterize the genome-wide miRNA expression profiles in Chinese Qinchuan bovine backfat and longissimus dorsi, and we sought to identify a panel of bovine miRNAs that could serve as novel biomarkers for cattle breeding programs.

## Materials and Methods

### Ethics statement

All animal protocols were approved by the Institutional Animal Care and Use Committee (IACUC) of Northwest A&F University and Meixian Qinbao Co., Ltd. All surgery was performed under sodium pentobarbital anesthesia, and all efforts were made to minimize suffering. The adult Qinchuan tissues were obtained from Meixian Qinbao Co., Ltd.

### Sample collection and RNA extraction

Qinchuan cattle are an outstanding traditional breed, known for its excellent beef production performance in China. Five adult Qinchuan cattle were randomly selected, and any two or more individuals with a traceable phylogenetic relationship were avoided in the sampling process. The adult Qinchuan backfat (AF) and longissimus dorsi (AM) were collected immediately after the animals were slaughtered, and were snap-frozen in liquid nitrogen until RNA extraction. Total RNA was extracted from the collected tissues using Trizol reagent (TaKaRa, Dalian, China). The RNA concentration and quality were further determined using the Agilent 2100 bioanalyzer (Agilent technologies, Santa Clara, CA, USA). The extracted RNAs from the same tissues were pooled prior to constructing the indexed libraries for Illumina sequencing technology (Beijing Genomics Institute, China).

### Small RNA sequence analysis

After clearing away the 3′ adaptor sequence, removal of redundancy and reads smaller than 18 nt, the clean reads were screened against and mapped to the latest bovine genome assembly (BosTau6, http://hgdownload.cse.ucsc.edu/goldenPath/bosTau6/bigZips/bosTau6.fa.gz) using the program SOAP [20]. To identify sequences originating from protein-coding genes, repeats, rRNA, tRNA, snRNA, and snoRNA, we used bovine mRNA (http://hgdownload.cse.ucsc.edu/goldenPath/bosTau6/database/refGene.txt.gz) and CDS (http://hgdownload.cse.ucsc.edu/goldenPath/bosTau6/bigZips/refMrna.fa.gz), RepeatMasker (http://www.repeatmasker.org) and Rfam data (v 10.1). Subsequently, the remaining reads were searched against the miRBase (version 19.0) to identify the conserved miRNAs. Only those small RNAs whose mature and precursor sequences perfectly matched known bovine miRNAs in miRBase were considered to be conserved miRNAs. To discover potential novel miRNA precursor sequences, unique sequences that have more than 10 hits to the bovine genome or match to other non-coding RNAs were removed. Then the flanking sequences (150 nt upstream and downstream) of each unique sequence were extracted for secondary structure analysis with Mfold (http://www.bioinfo.rpi.edu/applications/mfold) and then evaluated by Mireap (http://sourceforge.net/projects/mireap/). Those sequences residing in the stem region of the stem-loop structure and ranging between 20–22 nt with free energy hybridization lower than −20 kcal/mol were considered as novel miRNAs [21]. The filtered small RNA sequencing data were deposited in the National Center for Biotechnology Information Gene Expression Omnibus (http://www.ncbi.nlm.nih.gov/projects/geo/) under accession number GSE48569.

### MicroRNA expression analysis

Comparison of the known miRNAs expression between AF and AM was conducted to find out the tissue-enriched miRNAs. The procedures were shown as below: (1) Normalize the expression of miRNAs in AF and AM. Normalized expression (NE) = actual miRNA count/total count of clean reads. (2) Calculate fold-change and P-value from the normalized expression. Fold-change formula: Fold_change = log2 (AF NE/AM NE). P-value formula:
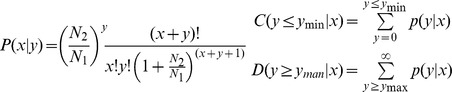
The x and y represented normalized expression levels, and the N1 and N2 represented total count of clean reads of a given miRNA in small RNA libraries of the AF and AM tissues, respectively. Stem-loop real-time reverse transcription polymerase chain reaction (RT-PCR) with SYBR Green was used for the validation of miRNA expression [22]. The bovine ribosomal protein S18 (RPS18) (GenBank NO. NM_001033614.1) gene was used as an endogenous control. The primers of tested miRNAs and the control gene were listed in Table S1. All reactions were carried out in triplicate. Analysis of relative miRNA expression data was used by the 2^−ΔΔCt^ method.

### Target gene prediction

A search for miRNA target genes was performed using the RNAhybrid software (http://bibiserv.techfak.uni-bielefeld.de/rnahybrid) and the rules used for target prediction from six aspects as followed: (1)No more than four mismatches between sRNA & target (G-U bases count as 0.5 mismatches); (2)No more than two adjacent mismatches in the miRNA/target duplex; (3)No adjacent mismatches in positions 2∼12 of the miRNA/target duplex (5′ of miRNAs); (4)No mismatches in positions 10 and 11 of miRNA/target duplex; (5)No more than 2.5 mismatches in positions 1∼12 of the miRNA/target duplex (5′ of miRNAs); (6)Minimum free energy (MFE) of the miRNA/target duplex should be more than or be equal to 75% of the MFE of the miRNA bound to it's perfect complement.

## Results

### Deep sequencing of bovine short RNAs

**Figure 1:**
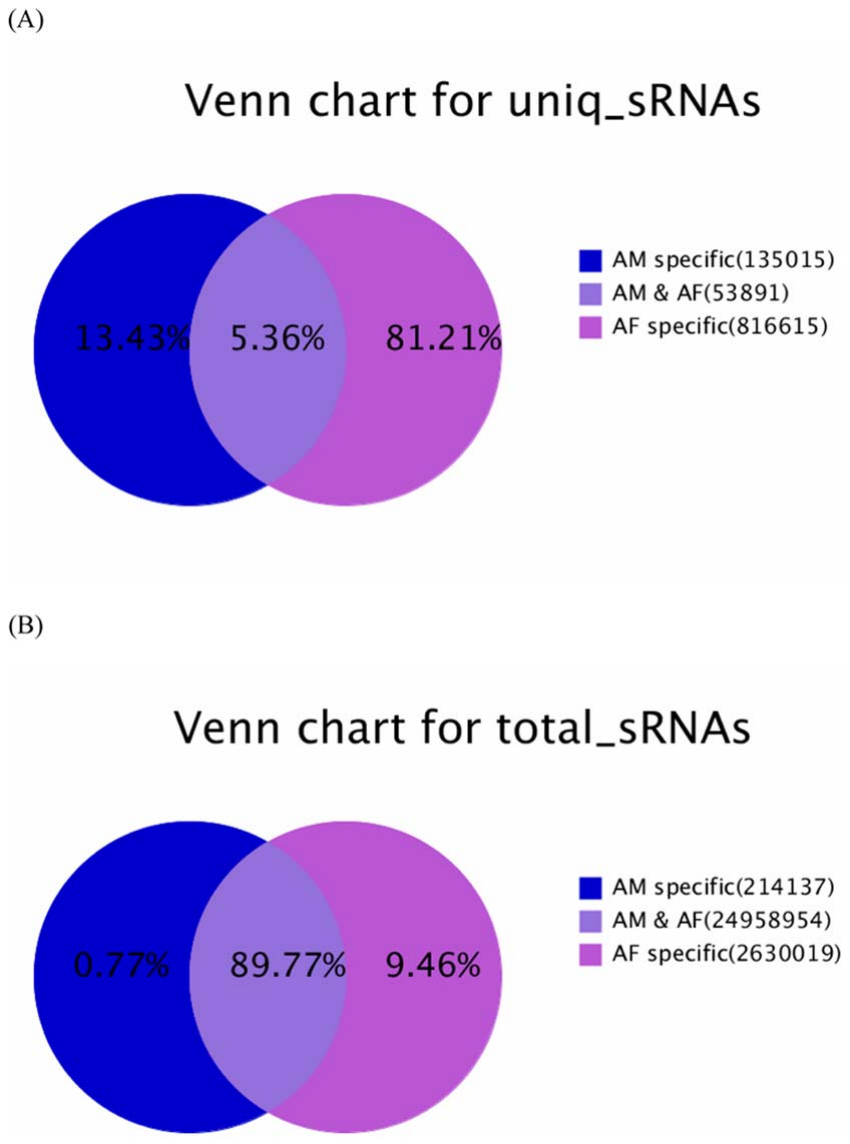
Summary of the common and specific tags of two samples, including the summary of unique tags (A) and total tags (B).

**Figure 2:**
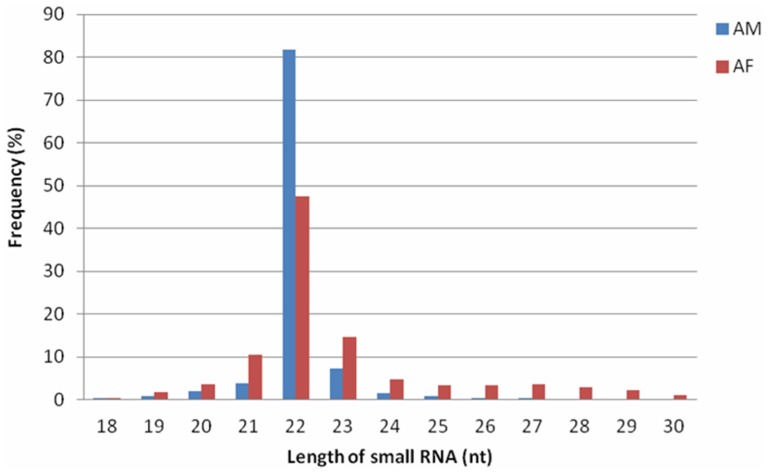
Length distribution of small RNAs in AF and AM libraries.

In order to identify novel and differentially expressed miRNAs between Qinchuan bovine AF and AM, two small RNA libraries were constructed for Illumina sequencing technology, and each library was pooled by five replicates. Sequencing provided a total of 14,373,930 and 13,641,806 reads of 18 nt–30 nt from AF and AM libraries, respectively. After removing the low quality, adaptor and insufficiently tags, a total of 14,244,946 and 13,558,164 clean reads were ultimately obtained (Table S2). The unique and total reads of common and tissue-specific small RNA tags in the two libraries were shown in Figure 1. The AF-specific unique sequences accounted for 81.21% of all sequencing reads and 13.43% in the AM library, respectively (Figure 1A). The percentages of the AF-specific and AM-specific sequences were 9.46% and 0.77% of the total small RNAs in the two libraries (Figure 1B). Length distribution analysis showed that most reads ranged from 21 to 23 nt. The percentage of the 22 nt reads in the total reads was 47.42% for the AF group and 81.86% for the AM group (Figure 2).

Next, all of the sequencing reads were aligned against the bovine genome (Btau_6.0) using the SOAP Program [20]. 9,690,034 reads were matched to the bovine genome in the AF library (250,668 unique sRNAs) and a total of 12,239,842 reads were presented in the AM library (100,581 unique sRNAs), respectively. Subsequently, the genome-matched small RNA sequences were clustered into several RNA classes such as known miRNAs, degraded fragments of mRNAs, repeats, rRNA, tRNA, snRNA/snoRNA and others (Table S3). Known miRNAs accounted for 47.89% of all sequencing reads in the AF library and 86.69% in the AM library, suggesting that mature miRNAs were highly enriched in our small RNA libraries. However, after analyzing the number of unique sequences, the proportion of small RNA sequences derived from known miRNAs represented only a very small fraction of the total number (0.44% and 1.39% in the AF and AM libraries). The highest fraction of unique sequences (64.46% and 49.33% in the two libraries) was unclassified small RNA sequences, which probably included novel miRNA candidates and other classes of regulatory RNAs.

### Identification of conserved bovine miRNAs

**Figure 3:**
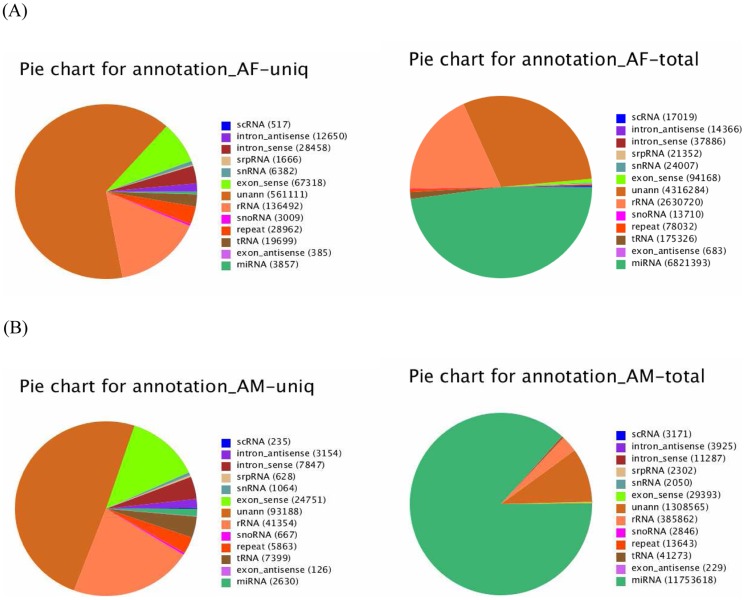
Distribution of the genome-mapped sequence reads in AF (A) and AM (B) small RNA libraries.

To identify conserved miRNAs in our dataset, all small RNA sequences with a length of 18–30 nucleotides were searched with Blastn against the known bovine mature miRNAs and their precursors in the miRNA database miRBase (http://www.mirbase.org). 3,857 unique sequences (6,821,393 reads) were annotated as miRNA candidates in the AF library as well as 2,630 unique sequences (11,753,618 reads) in the AM library (Figure 3). The miRNA candidates were then clustered into 412 and 334 categories corresponding to 442 and 361 independent genomic loci in the two libraries according to sequence similarity (Table 1), and only sequences with more than 10 reads in both libraries were shown (Table S4). Each category included multiple homologs, which differed in sequence length by only 1∼5 nucleotides. Such homologous sequences with different lengths are thought to be variants produced by various biochemical modifications and by imprecise processing of primary or precursor miRNAs by Drosha and Dicer enzymes.

**Table 1:** Summary of known miRNA in each sample.

	miR	miR-5p	miR-3p	pre-miRs	Unique matched to pre-miRs	Read matched to pre-miRs
Known miRs	598	79	78	766		
AF library	328	40	44	442	3926	6821671
AM library	261	38	35	361	2661	11753705

**Figure 4:**
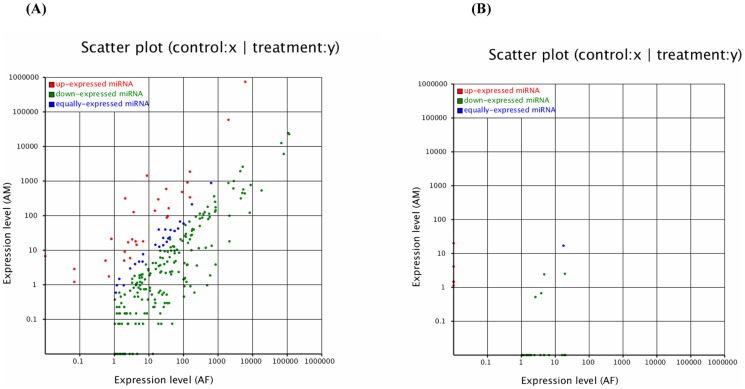
The differential expression of bovine conserved (A) and novel (B) miRNAs between AF and AM tissue were shown. Note: Expression level (AF): Expression level of adult bovine backfat tissue; Expression level (AM): Expression level of adult bovine muscle tissue. Each point in the figure represents a miRNA. Red points represent miRNAs with a fold change >2, blue points represent miRNAs with 1/2<fold change ≤2, green points represent miRNAs with fold change ≤1/2.

The expression profiles of each miRNAs between the two libraries were demonstrated in Figure 4A and Table S4. The results showed that 173 miRNAs at the expressed levels >10 reads consisted of 33 up-expressed miRNAs and 140 down-expressed miRNAs. Some miRNAs were significantly differentially expressed between the two libraries. For example, bta-miR-154c, -199a-3p, -320a and -432 were identified to be high expressed in bovine backfat, in contrast with the patterns shown by bta-miR-1, -133a, -378 and -206 in bovine muscle tissue. This suggested that these tissue-enriched miRNAs might affect the development of fat and/or muscle tissue.

### Validation of tissue-enriched miRNAs

**Figure 5:**
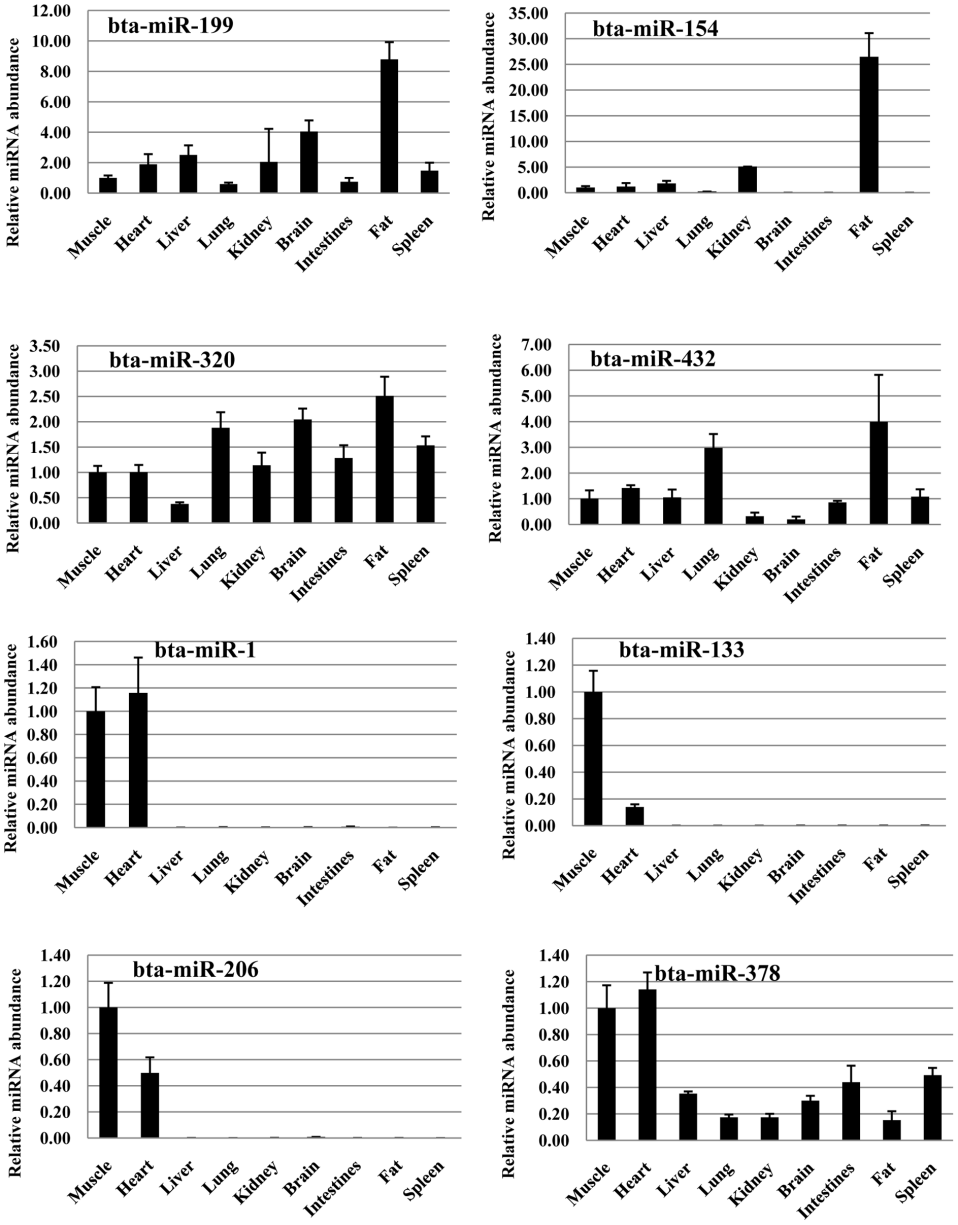
The expression of miRNAs in bovine different tissues were detected by RT-qPCR.

For validation and identification of tissue-enriched miRNAs in Qinchuan cattle, stem-loop qPCR [22] analysis of miRNA expression was performed in adult skeletal muscle, heart, liver, lung, kidney, brain, intestines, fat and spleen. In the present study, we picked out 8 significantly highly expressed miRNAs compared between AF and AM libraries, including bta-miR-199a-3p, -154c, -320a and -432 in backfat library and bta-miR-1, -133a, -206, and -378 in muscle library. The comparison between qPCR and deep sequencing showed that they were showing the same trends for all selected miRNAs. Comparison of miRNA expression profiles among tissues revealed that miR-154c in fat, and miRNA-1, miRNA-133 and miRNA-206 in muscle-related tissue or organs (skeletal muscle, heart) were specially expressed (Figure 5).

### Identification of novel bovine miRNAs

The characteristic hairpin structure of miRNA precursor can be used to predict novel miRNAs. We used a prediction software Mireap (http://sourceforge.net/projects/mireap/) to predict novel miRNA by exploring the secondary structure, the Dicer cleavage site and the minimum free energy of the unannotated small RNA tags which could be mapped to genome. Based on sequencing, we identified 27 novel bovine miRNAs, which corresponded to 39 genomic loci. A total of 22 miRNAs were in AF library and 10 were in AM library, of which 5 overlapped in both libraries (Table S5A). In addition, an examination of pre-miRNAs and other RNAs (tRNA, rRNA, and mRNA) revealed that miRNAs were significantly different from other RNAs [23]. In detail, more than 90% of miRNA precursors have a Minimal folding free energy indexs (MFEI) greater than 0.85, significantly higher than tRNAs (0.64), rRNAs (0.59), or mRNAs (0.65). The results suggested that the MFEI can easily be used to distinguish miRNA from other non-coding and coding RNAs. This provides a more precise criterion to predict miRNAs using computational approaches, and in our database 19 pre-miRNAs had a MFEI greater than 0.85. In addition, the expression profiles of the novel miRNA candidates by measuring the sequencing frequencies were showed in Figure 4B and Table S5B, and the results showed 27 novel miRNAs consisted of 5 up-expressed and 22 down-expressed miRNAs.

### Prediction of target genes

After the discovery of hundreds of miRNAs in AF and/or AM libraries, some miRNAs appeared to be expressed nearly in two tissues and others were specifically expressed at high numbers in one tissue. Remarkably, bta-miR-154c, bta-miR-199a-3p, bta-miR-320a and bta-miR-432 had a high number with 120128, 260952, 86405 and 31136 reads in AF library compared to 1601, 7158, 5832 and 244 reads in AM library, respectively. The contrastive patterns were shown by bta-miR-1, bta-miR-133a, bta-miR-378 and bta-miR-206 with 9799576, 18927, 24882 and 788710 reads in AM library as opposed to 89060, 127, 2209 and 28690 reads in AF library. In addition, stem-loop qPCR analysis also indicated that these miRNAs were fat/muscle-enriched compared to miRNAs expression in other tissues. The different expression patterns indicated that tissue-enriched miRNAs maybe have certain roles in the respective tissues. We therefore could postulate that bta-miR-154c, -199a-3p, -320a and -432 may influence lipid metabolism and adipogenesis in backfat tissue, and bta-miR-1, -133a, -378 and -206 may contribute to the regulation of muscle growth and phenotype. To identify the potential function of these miRNAs, target prediction was performed using RNAhybrid software (http://bibiserv.techfak.uni-bielefeld.de/rnahybrid). A total of 1109, 2024, 1937, 1693 and 1709 putative target sites for bta-miR-154c, -199a-3p, -320a, -432 and -378 in the present paper were identified (Table S6A, S7A, S8A, S9A and S10A), respectively. Next, all the target gene candidates were submitted for homology and annotation searches and Gene Ontology (GO) annotation using an online version of the Blast2GO program (www.Blast2GO.com). In total, 1109, 2024, 1937 and 1693 target sites of AF-enriched miR-154c, -199a-3p, -320a and -432 were annotated with 1380, 1300, 1793 and 708 GO terms (Table S6B, S7B, S8B and S9B), and 1709 GO terms (Table S10B) were associated to 1709 target sites of AM-enriched miR-378 in the Non-Redundant database. The distributions of GO term categories for the target genes of bta-miR-154c, -199a-3p, -320a, -432 and -378 were highly similar (Table S11). The biological functions identified by the Blast2GO program included categories related to a wide variety of physiological and biological events, such as cellular processes, metabolic processes, biological regulation, responses to stimulus, and multicellular organismal processes. Further analyses revealed that 370 AF-specific miRNA target genes relating to lipid metabolism and adipogenesis were annotated with 28 bta-miR-154c GO terms (Table S6C), 28 bta-miR-199a-3p GO terms (Table S7C), 47 bta-miR-320a GO terms (Table S8C) and 15 bta-miR-432 GO terms (Table S9C), respectively. And 61 target sites of AM-enriched bta-miR-378 were annotated within 27 GO terms (Table S10C).

Then, 370 AF-specific (Table S12A) and 61 AM-specific (Table S12B) target genes were investigated using Ingenuity Pathways Analysis software (IPA, www.ingenuity.com). Finally, the AF-specific genes were mapped to 25 genetic networks (Table S13A), and function of network 2, 3, 4, 5, 6, 8, 9, 10, 12, 13 and 15 were involved in lipid metabolism, respectively. A specific examination of the IPA molecular and cellular function revealed that 139 significantly different function annotations of the AF-specific targets relating to lipid metabolism were identified (Table S14A), and the top three biological functions were synthesis of lipid, concentration of lipid, fatty acid metabolism, respectively. Briefly, 142 molecules play a crucial role in synthesis of lipid, 110 in concentration of lipid as well as 103 in fatty acid metabolism. In addition, a total of 7 scored networks associated with AM-specific targets were detected. And network 2, having an IPA network score of 32 and 17 focus genes, presented function related to skeletal and muscular system development and function (Table S13B). Especially, 21 molecules were involved in development of muscle, 16 in muscle contraction and 16 in proliferation of muscle cells (Table S14B). Although the predicted targets were needed to be validated experimentally in the next experiments, these network and biological process analyses collectively illustrated some of the possible roles of the differential highly expressed miRNAs between AF and AM.

## Discussion

The cattle, a major source of meat-based protein, is an economically important livestock animal. Bovine muscle is a tissue of major economic importance for meat production. Recently, special attention has been paid to double-muscled cattle, and their muscles contain twice the number of fibers [24]. But their meat is less tasty due to the lower fat content. Thus in some countries, the production of fat animals is favored to ensure the production of marbled beef, which is rich in fat and has a high economic value [25]. Marbling has been shown to play an important role in the eating quality and composition of meat [26]. To better understand the biological mechanisms between AF and AM that may improve fat content in beef animals, the adult Chinese Qinchuan bovine AF and AM tissues were thus collected and two pooled miRNA libraries were constructed for Next-generation sequencing (NGS).

Of the mappable sequences, the majority of the small RNAs were 21∼24 nt in size, which coincided with the known specificity for Dicer processing and the features of mature miRNAs [27]. In the present study, the 22 nt sequences in AF and AM were the dominant small RNAs. This was in agreement with Chen et al., who reported that 21∼23 nt sequences was significantly greater than others, and almost half of the sequences in the backfat of Large White and Meishan pigs were canonical 22 nt miRNA [28]. However, our findings were not consistent with the previous studies on bovine testis and ovaries [29] and even of those in maize [30]. The differences observed between our study and previous studies may have been due to different experimental approaches (NGS vs random cloning), different species (cattle vs maize), and/or different tissues (AF and AM tissues vs testis and ovary tissues).

In our datasets, bta-miR-199a-3p showed the highly abundance followed by bta-miR-154c, bta-miR-320a and bta-miR-432 in AF library compared to AM library, respectively. Bta-miR-199a-3p was firstly cloned from the bovine adipose tissue [31], and the putative hairpin precursor resides in chromosome 16 in the bovine genome. In the present study, bta-miR-199a-3p exhibited a 5.12-fold significant increase in AF tissue compared with AM tissue. And it has been widely studied due to its possible involvement in tumor progression. Our finding showed that a total of 2024 putative conserved targets were identified for the bta-miR-199a-3p. These target genes belonged to 1300 GO terms, including lipid metabolic process, lipid biosynthetic process and fatty acid metabolic process, etc. These results indicated that bta-miR-199a-3p may be involved in adipose tissue development in beef. Our finding was consistent with the previous finding in the human mesenchymal stromal cells (hMSCs) [32]. MiR-199a regulates the expression of fatty acid binding protein 4 (FABP4) during adipogenic for hMSC differentiation. Bta-miR-154c was determined in the reproductive organs of Holstein cattle, and it appeared to be bovine-specific [29]. We retrieved 1109 predicted targets of miR-154c, and loaded them onto the Blast2GO program. We were then able to identify 28 GO terms relating to lipid metabolism and adipogenesis. Bta-miR-320a comprised of 2 isoforms: miR-320a-1 located on bovine chromosome 8 from 70060384 to 70060465 and miR-320a-2 located on bovine chromosome 20 from 15213924 to 15214005 [33]. MiR-320a has also been shown to involve in the MAPK and Wnt pathways [34], which are related to energy metabolism through modulating lipid metabolism [35], [36]. Bta-miR-432 was initially identified by computer analysis of other animal sequences by Artzi and colleagues in 2008 [37]. Currently, function of miR-432 in adipose tissue development has not been determined. To gain further insights into the latter function of AF-enriched miRNAs, we performed IPA and identified several gene networks of their target sites that were highly enriched in lipid metabolism. In network 2, bta-miR-320a and -432 targeted adenosine monophosphate-activated protein kinase alpha 1 (PRKAA1) and bta-miR-199a-3p targeted PRKAA1/2, which are the master regulators in glucose and lipid metabolism [38]. This may be similar to the results between miR-33 and PRKAA1. Over-expression of miR-33 in hepatocytes down regulates expression of PRKAA1, decreasing β-oxidation of fatty acids and increasing triglyceride accumulation [39]. Additionally, bta-miR-320a and -432 targeted peroxisome proliferator-activated receptor-gamma (PPARG) in network 5, and bta-miR-320a targeted peroxisome proliferator-activated receptor-alpha (PPARA) in network 8, respectively. Recently, PPARG has been studied as transcription factor involved in adipocyte differentiation and the functional potential to improve intramuscular fat deposition [40], while PPARA has been shown to regulate obesity by both increasing hepatic fatty acid oxidation and decreasing the levels of circulating triglycerides responsible for adipose cell hypertrophy and hyperplasia [41]. Hence, we also can assess that these AF-enriched miRNAs are likely to play roles in the development of bovine backfat tissues.

In this paper, the expression levels of the bta-miR-1, -133a, -206 and -378 in the muscle were higher than the backfat, which indicated a role for these miRNAs in the muscle. Previous studies have shown that miR-1, miR-133, and miR-206 can target multiple muscle development related genes. Specifically, miR-1 promotes myogenesis by targeting HDAC4, a transcriptional repressor of muscle gene expression. In contrast, miR-133 enhances myoblast proliferation by repressing SRF [42]. Also, miR-1 and miR-206 regulate Pax7 directly. Inhibition of these two substantially enhances satellite cell proliferation and increases Pax7 protein levels *in vivo* [43]. The consistent expression pattern and high conservation indicated that bta-miR-378 was also likely to play the similar roles in the development of bovine muscle tissues. Additionally, recent results indicated that microRNA-378 can regulate nephronectin expression modulating osteoblast differentiation by targeting GalNT-7 [44]. Our data showed that a total of 1709 target sites of bta-miR-378 belonged to 1709 GO terms, and 61 target genes relating to skeletal and muscular system development and function were annotated within 27 GO terms. To better understand the functions of the bta-miR-378 target sites, IPA analysis was performed and several gene networks were identified. In network 2, bta-miR-378 targeted cysteine and glycine-rich protein 3 (CSRP3). CSRP3 (coding for CRP3) is expressed only in striated muscle and its expression coincides with myogenic differentiation [45]. The nuclear CRP3 serves as a cofactor for the myogenic basic helix-loop-helix (bHLH) proteins (MyoD, MRF4, and myogenin), by promoting their interaction with the E-Box elements in the regulatory region of most muscle specific genes [46]. Therefore, we hypothesized that bta-miR-378 may contribute to muscle development and differentiation by targeting *CSRP3* gene in beef cattle and that bta-miR-378 could be a target for cattle breeding programs.

## Conclusions

We have identified 412 known and 22 novel miRNAs in backfat, and 334 known and 10 novel miRNAs in longissimus thoracis in Chinese Qinchuan beef cattle using deep sequencing technology. This study expands the repertoire of bovine miRNAs and could initiate further study in the fat and muscle development of beef cattle. In addition, bta-miR-199a-3p, -154c, -320a and -432 in backfat tissue, as well as bta-miR-1, -133a, -206, and -378 in muscle tissue, were significantly highly expressed, suggesting that they were likely to play roles in the development of bovine fat and/or muscle tissues and could be potential molecular markers for bovine genetics and breeding. Putative targets for these tissues-enriched miRNAs were predicted and 370 AF-specific and 61 AM-specific target genes were identified using the Blast2GO program. The relationship between target genes and these tissues-enriched miRNAs may be a novel pattern and could serve as a new biomarker for cattle breeding programs.

## Supporting Information

Table S1 Stem-loop RT-PCR Primer.
(XLS)

Table S2 Summary of small RNA sequencing date in backfat (A) and longissimus dorsi (B) libraries.
(XLSX)

Table S3 Distribution of the genome-mapped sequence reads in AF (A) and AM (B) small RNA libraries.
(XLSX)

Table S4 The known miRNAs expression profiles between two libraries.
(XLSX)

Table S5 **A** Novel miRNAs identified in this study. **B** The novel miRNAs expression profiles between two libraries.
(XLSX)

Table S6 Predicted targets for bta-miR-154c.
(XLS)

Table S7 Predicted targets for bta-miR-199a-3p.
(XLSX)

Table S8 Predicted targets for bta-miR-320a.
(XLSX)

Table S9 Predicted targets for bta-miR-432.
(XLSX)

Table S10 Predicted targets for bta-miR-378.
(XLSX)

Table S11 GO term distribution. Standard configuration of the Blast2GO web application (http://www.blast2go.de) was applied to generate level 2 graphs for GO-term distributions to the biological process.
(DOC)

Table S12 Description of the tissue-specific target genes.
(XLS)

Table S13 Ingenuity path analysis (IPA) gene network of tissue-specific target sites.
(XLSX)

Table S14 Description of the molecular and cellular biological functions significantly modulated within the tissue-specific genes.
(XLSX)
