# Supplementary material for: Comparative Transcriptome Analysis Reveals Significant Differences in MicroRNA Expression and Their Target Genes between Adipose and Muscular Tissues in Cattle
Source: PLoS One. 2014 Jul 9;9(7):e102142. doi: 10.1371/journal.pone.0102142 (PMC4090223; doi:10.1371/journal.pone.0102142)
Supplement: Table S11 — GO term distribution. Standard configuration of the Blast2GO web application (http://www.blast2go.de) was applied to generate level 2 graphs for GO-term distributions to the biological process. (DOC) [file pone.0102142.s011.doc]

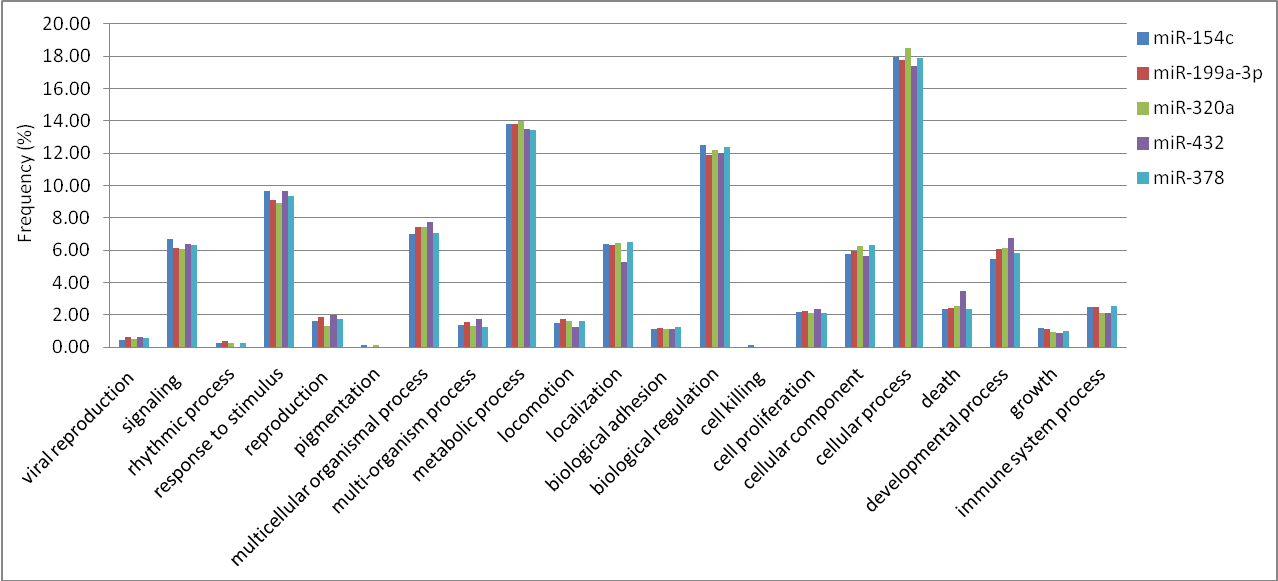


**Table S11** GO term distribution. Standard configuration of the Blast2GO web application ([http://www.blast2go.de](http://www.blast2go.de/)) was applied to generate level 2 graphs for GO-term distributions to the biological process.
